# Mental preparation in runners: gender differences, competition levels, and psychological training effects on performance

**DOI:** 10.3389/fspor.2024.1456504

**Published:** 2024-11-15

**Authors:** Bence Kelemen, Renátó Tóth, Ottó Benczenleitner, László Tóth

**Affiliations:** ^1^ School of Doctoral Studies, Hungarian University of Sports Science, Budapest, Hungary; ^2^ Institute of Sports Sciences, Eszterházy Károly Catholic University, Eger, Hungary; ^3^ Department of Psychology and Sport Psychology, Hungarian University of Sports Science, Budapest, Hungary; ^4^ Teacher Training Institute, Hungarian University of Sports Science, Budapest, Hungary

**Keywords:** distance running, sport psychology, mental training, mental skills, race tactics

## Abstract

**Introduction:**

The present study aimed to investigate the mental and sports psychological preparation, as well as tactical preparation, of distance runners for competition. We examined whether there are differences based on gender, competition level and various race disciplines, as well as how mental preparation influences sports skills applicable in different competitive situations.

**Methods:**

The sample consisted of 201 distance runners who completed the Sports Mental Training Questionnaire (SMTQ) alongside assessments of their sports psychology and race tactics.

**Results:**

The results indicated that neither gender, competition level, nor race discipline had a significant impact on mental preparedness. However, women demonstrated notably higher scores in the use of self-talk as a mental technique. Additionally, participants who received training in sports psychology scored significantly higher across several mental skills, as well as on the overall mental preparedness score.

**Discussion:**

This article validates the SMTQ and its association with mental readiness, as confirmatory factor analysis demonstrates adequate validity. Additionally, mental preparation was found to enhance performance and well-being among distance runners. Further research is needed to explore the impact of group interventions to broaden the reach of mental training programs.

## Introduction

Achieving peak performance in sports is a complex, multidimensional process that involves the integration of various disciplines and factors. The optimization of human performance necessitates a thorough and methodical evaluation aimed at identifying both facilitators and potential barriers to success. The three primary domains of sports preparation encompass physical and technical training, tactical development, and mental or sports psychology-based preparation (1). Each of these elements plays a critical role in shaping overall performance outcomes, requiring a holistic approach to reach the highest levels of athletic achievement (2). The emphasis on scientific studies concerning physical preparation has often overshadowed the equally vital aspects of tactical strategy and mental preparation, despite their considerable impact on performance outcomes in endurance sports. This research aims to address this gap by specifically focusing on the mental preparation of distance runners.

Since the 1970s, researchers and coaches have identified the physiological factors that best predict distance running performance, specifically running times, including maximum oxygen uptake (VO_2_ max), associated speed (vVO_2_ max), velocity at lactate threshold (vLT_2_), and running economy (RE) (3–5). Established protocols exist for the testing and monitoring of these physiological variables (6–8), and training plans designed to enhance these factors are increasingly grounded in scientific principles. These areas of focus have garnered considerable attention in both academic literature and coaching practice. Distance runners cover 120–180 km per week at their highest level, while marathon runners can average 200–220 km per week (9–11). This consists of 11–14 weekly running sessions but often includes strength training in a gym. The training of modern distance runners is characterized primarily by an emphasis on aerobic capacity development (12, 13). Training methods now include systematically developing anaerobic threshold speed (vLT_2_), mainly through interval training, using lactate measurement and heart rate monitoring to maintain appropriate intensity zones (14). Since the 2010s, two prominent endurance training methods have emerged: the Norwegian double-threshold and Stephen Seiler's polarized training (12). The Norwegian method combines a high volume of aerobic running with four interval sessions weekly, performed at intensities between the aerobic (vLT1) and anaerobic thresholds (vLT_2_) (15–17). Seiler's polarized model uses an “80:20” approach, where 80% of training is low-intensity, and 20% consists of two higher-intensity sessions—one longer near vLT_2_ and one shorter, faster (18). Both methods include race-specific fatigue simulations in the final 1–2 months before key competitions (11).

In distance running, different tactical scenarios—such as record-breaking strategies in Grand Prix races and winning tactics in national and global championships—demand varied responses from athletes to achieve success (19–24). During international and national championships, the distribution of effort through multiple rounds and different changes of pace largely determine the fate of medals. In longer race distances (marathons and ultramarathons), the right pace and the ability to cope with physical and mental fatigue (e.g., the so-called “marathon wall”) are critical to completing the distance successfully (25–27). Due to the high stakes, the unpredictable behavior of the runners and the high pain levels, these events require complex mental skills from the runners (24, 28, 29). As summed up by successful Polish running coach Tomasz Lewandowski: “A championship is not like a Gold meet or the Diamond League, where you have pacers, and you go after them, or you follow the wavelight. A championship is something different; it is about energy distribution over a few rounds, it is about tactics, and it is about reading the race. It is really about performance, not about time trials. It is completely different” (30).

While tactical analyses, physiological training and testing have a strong tradition and a scientifically based system in distance running, the mental and psychological aspects of the sport have only started to catch up in recent decades. Most of the literature on distance runners focuses on the motivation of recreational runners and the impact of running on mental well-being (31–34). There are relatively few studies on higher-level national or international competitors, and those that exist are more recent, primarily addressing the general psychological traits of these athletes (35, 36). The significance of mental preparation raises important questions in several respects. Historical examples abound of runners who achieve outstanding season-best performances (37), yet struggle to replicate these results in world competitions, or fail to translate their training success into competitive settings due to anxiety, commonly referred to in the literature as “choking” (38–40). Recent research and practical insights indicate that various psychological factors, as outlined in the Central Governing Model, contribute to fatigue, and optimizing these factors can lead to improved performance outcomes (41). Research into the core elements of mental training is ongoing (42, 43), and understanding these elements is critical for effective psychological assessment and preparation for sports performance. Behnke and colleagues drew on previous work to identify the most vital factors: Foundation Skills, Performance Skills, Interpersonal Skills, and mental techniques. Based on these factors, they developed the Sport Mental Training Questionnaire (SMTQ), a psychological assessment tool (44). Self-talk and mental imagery were considered the most typical mental techniques athletes use (45, 46). Mental training has been based on the assumption that psychological factors enhance or inhibit physical performance (47). Stress interferes with cognitive focus on the task and increases self-focus, leading to lower levels of sports performance (48, 49). Excessive stress and the regulation of negative emotions can enhance performance (50, 51). The general theoretical rationale for using mental training is to equip athletes with new skills that effectively foster a stronger mindset. For example, this includes utilizing mental resources to reduce negative states, such as anxiety through positive self-talk (52), or enhancing positive states through relaxation routines that improve focus (53). Several components of mental training have been validated, e.g., mental imagery techniques (54), self-talk techniques (55) and pre-performance routines (56).

### Research aims

The present study seeks to address a gap in the literature on mental preparation in sports. Our primary research question investigates whether the 20-item English-language Sports Mental Training Questionnaire (SMTQ) serves as a brief and valid measure of mental preparedness in distance runners. Additionally, we aim to examine potential differences across genders, competition levels, and various race disciplines. We also explore how mental preparation impacts the sports skills applicable in diverse competitive situations. Ultimately, this research aims to enhance scientific understanding of the mental, psychological, and tactical preparation of distance runners for competition while evaluating a practical measurement tool for use in coaching contexts.

## Material and methods

### Participants and procedure

The sample comprised 201 athletes (110 men, 54.7%, and 91 women, 45.3%) invited to complete an online questionnaire via email through various sports clubs. During recruitment, we informed athletes that the language of the study was English; therefore, only athletes with at least an advanced level of English participated. The mean age for the male participants was 35.05 years (*SD* = 12.57), while the mean age for female participants was 35.98 years (*SD* = 10.87). Participants included recreational athletes (*n* = 112), national-level athletes (*n* = 56), and international-level athletes (*n* = 33). The athletes competed in a range of running events, including middle-distance running (800–1,500 m; *n* = 44), long-distance running (5,000–10,000 m; *n* = 36), half marathon and marathon (*n* = 79), and ultra running (distances longer than 42.2 km; *n* = 42). The respondents hailed from multiple countries, with the highest representation from Hungary (*n* = 71), followed by the United States (*n* = 33), the United Kingdom (*n* = 29), Italy (*n* = 14), Australia (*n* = 14), and New Zealand (*n* = 11), as well as smaller numbers from Kenya (*n* = 7), Norway (*n* = 4), Sweden, Canada, and Germany. The study protocol was reviewed and approved by the Hungarian University of Sport Science Research Ethics Committee, and written informed consent was obtained from all participants prior to the study.

### Measures

Participants were initially prompted to provide demographic data, including their age, nationality, competition level (categorized as recreational, national, or international), and race discipline (middle distances; long distances; half marathon, and marathon; ultra distances). Subsequently, they responded to questions pertaining to both mental and race-specific tactical preparation. To assess mental preparation, they were asked: “Do you incorporate any mental preparation into your training process?” This was a straightforward binary (yes/no) question. Based on their responses, participants were divided into two groups: those who engaged in mental preparation, referred to as the Mental Preparation (M.P.) group, and those who did not engage in such techniques, categorized as the No Mental Preparation (N.M.P.) group. For those who answered affirmatively, additional inquiries probed the context in which mental preparation was utilized, as well as the specific techniques employed. Respondents were permitted to select multiple techniques from the options provided. Concerning tactical preparation, the following question was posed: “How do you prepare for the tactical aspects of a race, such as adjusting pace or responding to the strategies of potential opponents?” Again, participants were allowed to select multiple responses to capture the breadth of their tactical approaches.

Mental skills in sports were measured using a validated sport psychology questionnaire, Sport Mental Training Questionnaire (SMTQ) (44), administered in English. This 20-item inventory provided an overall mental training score, as well as scores for five subscales: Foundational Skills (F.S.), Performance Skills (P.S.), Interpersonal Skills (I.S.), and mental techniques, including Self-Talk (S.T.) and Imagery (I.M.). Participants responded using a 5-point Likert scale (1 = “strongly disagree”, 5 = “strongly agree”). Example items from the questionnaire included statements such as: “I am able to “bounce back” and overcome any failure; it does not discourage me from further action” (Foundational Skills); “During a competition, I am able to adapt quickly to changes in the performance situation and to distracting factors” (Performance Skills); “I know and I follow the rules established in the training group” (Interpersonal Skills); and “Before the start, I rehearse my performance in my mind, imagining it exactly as I want it to unfold during the actual competition” (Imagery).

### Statistical data analyses

Normality was checked for each measurement, and all showed a normal distribution. Descriptive statistics and Pearson's intercorrelations were then calculated. We calculated point-biserial correlations to examine the relationship between gender and various psychological skills, assessing whether gender influences these performance factors among athletes. Gender and groups doing and not doing mental training were compared using an independent samples *t*-test, while participation level and different competitive events were compared using one-way ANOVA. Confirmatory factor analysis (CFA) was performed to test the instrument's construct (factor) validity, which, in our case, is the SMTQ. There are no uniform guidelines for confirmatory factor analysis on which goodness-of-fit indicators better predict the model under study. Therefore, it is recommended to use a combination of indicators in the Analysis (57). In the present study, the chi-square to the degree of freedom ratio (χ^2^/pdf), root mean square error of the mean square error (RMSEA), standardized root mean square residual (SRMR) and comparative fit index (CFI) were used, to interpret confirmatory factor analysis. For the χ^2^/df index, there is no generally accepted cut-off value, and we follow the suggestion of (57) that a value lower than 3 is acceptable. For the RMSEA and SMR indices, a value lower than 0.08 is acceptable. In their earlier study, Hu and Bentler (58) found that a CFI value above 0.90 is acceptable, but later, based on their analyses, van Laar and Braeken suggested that a value above 0.95 should be considered acceptable (59). To explore the reliability, an internal consistency test was performed, with Cronbach's alpha coefficient greater than 0.60 considered acceptable (60). In addition, McDonald's omega was also calculated to assess the internal consistency of the subscales using JAMOVI 2.4.11 software (61, 62). For all statistical analyses, we used IBM SPSS version 27, except for the CFA, where we used JAMOVI version 2.4.11. In the case of the CFA, we used Full Information Maximum Likelihood (FIML) estimation. During the analyses no error terms between items were allowed to correlate.

## Results

### Descriptive statistics and intercorrelations

Before running the primary analyses, we checked the normal distribution of the sample, which shows that for all variables, the skewness is between −2 and 2, the peak is between −7 and +7, and there are no extreme outliers. This suggests that the normal condition is fulfilled, so robustness tests are unnecessary (63, 64). All subscales and the total values of the scales showed internal consistency values of acceptable (*α* > 0.70) and sound (*α* > 0.80), respectively (65). McDonald's Omega scores ranged from 0.69 to 0.95. Scores below 0.6 are not acceptable, but McDonald's Omega scores above 0.7 and 0.8 can be interpreted as good estimates of internal consistency (66). All subscales showed a close significant positive correlation (*p* < 0.001) with each other. The point-biserial correlation between gender and self-talk (*r* = 0.179, *p* = 0.011) indicates a significant, positive relationship, suggesting that women tend to report higher self-talk scores than men, while no other significant positive correlations were found between gender and the remaining variables. The descriptive statistics, reliability and correlation matrix of the scales are presented in Table 1.

**Table 1:** Descriptive statistics.

	M	SD	*α*	*ω*	Skewness	Kurtosis	Age	FS	PS	IS	ST	IM	TOT
Age	35.47	11.82			0.36	−0.87							
FS	14.76	2.85	0.70	0.69	−0.58	0.75	−.030						
PS	21.93	4.07	0.85	0.79	−0.46	0.54	.050	.692^b^					
IS	15.02	3.03	0.76	0.76	−0.89	1.62	−.130	.226^b^	.314^b^				
ST	12.04	2.60	0.87	0.85	−1.19	1.37	−.110	.325^b^	.325^b^	.243^b^			
IM	10.32	2.86	0.77	0.89	−0.54	−0.15	−.110	.311^b^	.355^b^	.290^b^	.461^b^		
TOT	74.05	10.83	0.86	0.95	−0.73	2.14	−.080	.747^b^	.818^b^	.592^b^	.638^b^	.671^b^	

FS, foundation skills; PS, psychological skills; IS, interpersonal skills; ST, self-talk; IM, imaginary; TOT, total score.

^a^ Correlation is significant at the 0.05 level (2-tailed).

^b^ Correlation is significant at the 0.01 level (2-tailed).

### Results of confirmatory factor analysis

**Figure 1:**
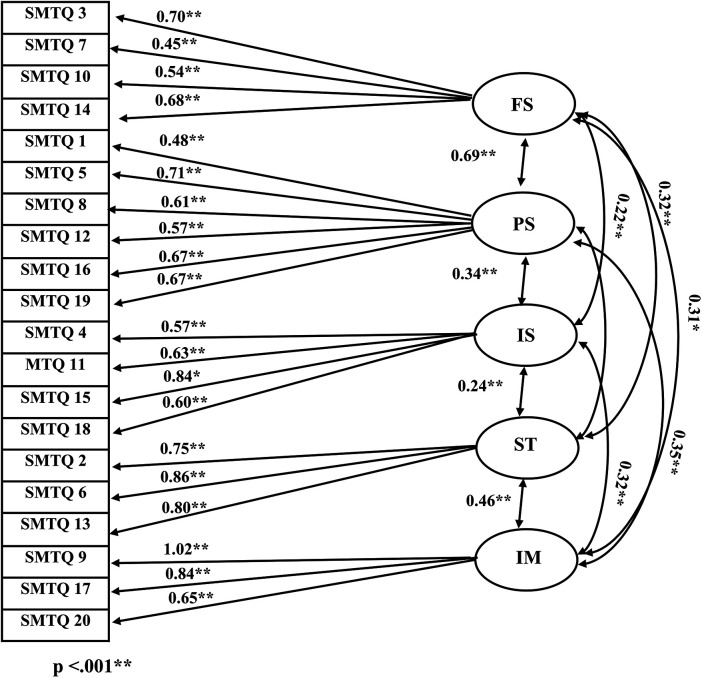
Item and factor loadings of the Sports Mental Training Questionnaire (SMTQ).

The results of the confirmatory factor analysis indicate a significant model (*p* < 0.001), with values [χ^2^(246) = 160, CFI = 0.95, TLI = 0.94, SRMR = 0.06, RMSEA = 0.05], confirming the construct validity of the 20-item English-language Sports Mental Training Questionnaire (SMTQ) (see Table 2). The factor loadings of all items were examined in the Analysis, all of which show significant results (*p* < 0.001) and are shown in Table 3. Figure 1 illustrates the factor loadings of the items and the correlations between the scales, which are significant in all cases (*p* < 0.001). Our results suggest that the structure and reliability of the SMTQ factor structure are as predicted.

**Table 2:** Results of confirmatory factor analysis (CFA).

	χ^2^	df	*p*	CFI	TLI	SRMR	RMSEA
SMTQ	246	160	<0.001	0.95	0.94	0.06	0.05
Acceptable value	χ^2^/pdf > 3		<0.05	>0.95	>0.90	<0.08	<0.08

**Table 3:** Factor loadings.

Factor	Indicator	Estimate	S.E.	*Z*	*p*
FS	SMTQ3	0.706	0.0725	9.74	<.001
	SMTQ7	0.453	0.0647	7.00	<.001
	SMTQ10	0.542	0.0610	8.89	<.001
	SMTQ14	0.689	0.0755	9.13	<.001
PS	SMTQ1	0.487	0.0548	8.89	<.001
	SMTQ5	0.712	0.0598	11.90	<.001
	SMTQ8	0.618	0.0550	11,25	<.001
	SMTQ12	0.576	0.0588	9.80	<.001
	SMTQ16	0.673	0.0597	11.26	<.001
	SMTQ19	0.679	0.0573	11.87	<.001
IS	SMTQ4	0.573	0.0752	7.62	<.001
	SMTQ11	0.636	0.0604	10.52	<.001
	SMTQ15	0.846	0.0658	12.86	<.001
	SMTQ18	0.600	0.0730	8.22	<.001
ST	SMTQ2	0.757	0.0578	8.13	<.001
	SMTQ6	0.866	0.0572	15.14	<.001
	SMTQ13	0.805	0.0598	13.47	<.001
IM	SMTQ9	1.027	0.0838	12.26	<.001
	SMTQ17	0.847	0.0771	10.99	<.001
	SMTQ20	0.659	0.0764	8.63	<.001

FS, foundation skills; PS, psychological skills; IS, interpersonal skills; ST, self-talk; IM, imaginary.

### Differences between groups

The one-way ANOVA results indicated that there were no statistically significant differences between runners specializing in different distances (Middle-distance vs. Long-distance vs. Half-marathon and Marathon vs. Ultramarathon) across all the measured skills. For Foundation Skills, no significant difference was found [*F*(3, 197) = 0.268, *p* = 0.848]. Similarly, Performance Skills showed no significant difference [*F*(3, 197) = 0.510, *p* = 0.676]. The same was true for Interpersonal Skills [*F*(3, 197) = 0.820, *p* = 0.484]. For Self-Talk, the results were also not significant [*F*(3, 197) = 0.610, *p* = 0.609]. Imaginary Skills showed no significant differences either [*F*(3, 197) = 0.801, *p* = 0.495]. Finally, for the overall Total score, the ANOVA did not reveal any significant differences [*F*(3, 197) = 0.092, *p* = 0.964].

The results of the ANOVA analysis indicate that there were no statistically significant differences among the groups based on the competition level (Recreational Level vs. National Level vs. International Level). For Foundation Skills, the analysis yielded *F*(2, 198) = 1.019, *p* = 0.363, suggesting no significant differences between groups. Similarly, Performance Skills showed *F*(2, 198) = 1.882, *p* = 0.155, indicating that any differences in means were not statistically significant. The Interpersonal Skills category approached significance with *F*(2, 198) = 2.743, *p* = 0.067, but did not reach the conventional threshold for significance. Self-Talk exhibited no significant differences as well, *F*(2, 198) = 0.162, *p* = 0.851. Imaginary skills and the overall total also reflected non-significant results, with *F*(2, 198) = 0.542, *p* = 0.582 and *F*(2, 200) = 1.289, *p* = 0.278, respectively. Overall, the findings indicate that the skill categories assessed do not differ significantly among the groups analyzed.

Furthermore, no significant differences were found when comparing low-level (Recreational level) and higher-level (National and International level) groups as reported in the literature (67, 68) using independent samples *t*-tests. For Foundation Skills, assuming equal variances, no significant difference was found [*t*(199) = 0.383, *p* = 0.702], with a mean difference of 0.156 (95% CI: −0.644–0.956). For Performance Skills, equal variances were assumed, and no significant difference was observed [*t*(199) = −0.443, *p* = 0.658], with a mean difference of −0.256 (95% CI: −1.397–0.884). In the case of Interpersonal Skills, there was a marginal significance when equal variances were assumed [*t*(199) = 1.972, *p* = 0.050], with a mean difference of 0.840 (95% CI: 0.000–1.680). For Self-Talk, the test indicated no significant difference [*t*(199) = 0.567, *p* = 0.571], with a mean difference of 0.210 (95% CI: −0.519–0.938). Similarly, for Imaginary Skills, no significant difference was found [*t*(199) = 0.986, *p* = 0.325], with a mean difference of 0.400 (95% CI: −0.400–1.201). Finally, the Total score did not show significant differences [*t*(199) = 0.878, *p* = 0.381], with a mean difference of 1.350 (95% CI: −1.681–4.380).

When comparing those who performed mental preparation as part of the training process (M.P., *n* = 136) with those who did not (N.M.P., *n* = 65), the Independent Samples *t*-test revealed statistically significant differences between the groups for several skills. For Foundation Skills, no significant difference was found [*t*(199) = 0.324, *p* = 0.747], with a mean difference of 0.140 (95% CI: −0.711–0.990). Similarly, for Performance Skills, there was no significant difference [*t*(199) = −0.202, *p* = 0.840], with a mean difference of −0.124 (95% CI: −1.337–1.088). However, for Interpersonal Skills, a significant difference was observed [*t*(199) = −2.610, *p* = 0.010], with a mean difference of −1.174 (95% CI: −2.061 to −0.287). The non-preparation group (*M* = 14.23, *SD* = 3.18) scored lower than the preparation group (*M* = 15.40, *SD* = 2.88). The Self-Talk dimension also showed a significant difference [*t*(199) = −3.303, *p* = 0.001], with a mean difference of −1.264 (95% CI: −2.018 to −0.509). The non-preparation group (*M* = 11.18, *SD* = 3.15) scored lower than the preparation group (*M* = 12.45, *SD* = 2.18). In the case of Imaginary Skills, a significant difference was found [*t*(199) = −3.180, *p* = 0.002], with a mean difference of −1.342 (95% CI: −2.174 to −0.510). The non-preparation group (*M* = 9.42, *SD* = 2.88) again scored lower than the preparation group (*M* = 10.76, *SD* = 2.75). Lastly, the Total score revealed a significant difference [*t*(199) = −2.331, *p* = 0.021], with a mean difference of −3.764 (95% CI: −6.949 to −0.579). The non-preparation group (*M* = 71.51, *SD* = 11.32) scored lower than the preparation group (*M* = 75.27, *SD* = 10.40).

**Figure 2:**
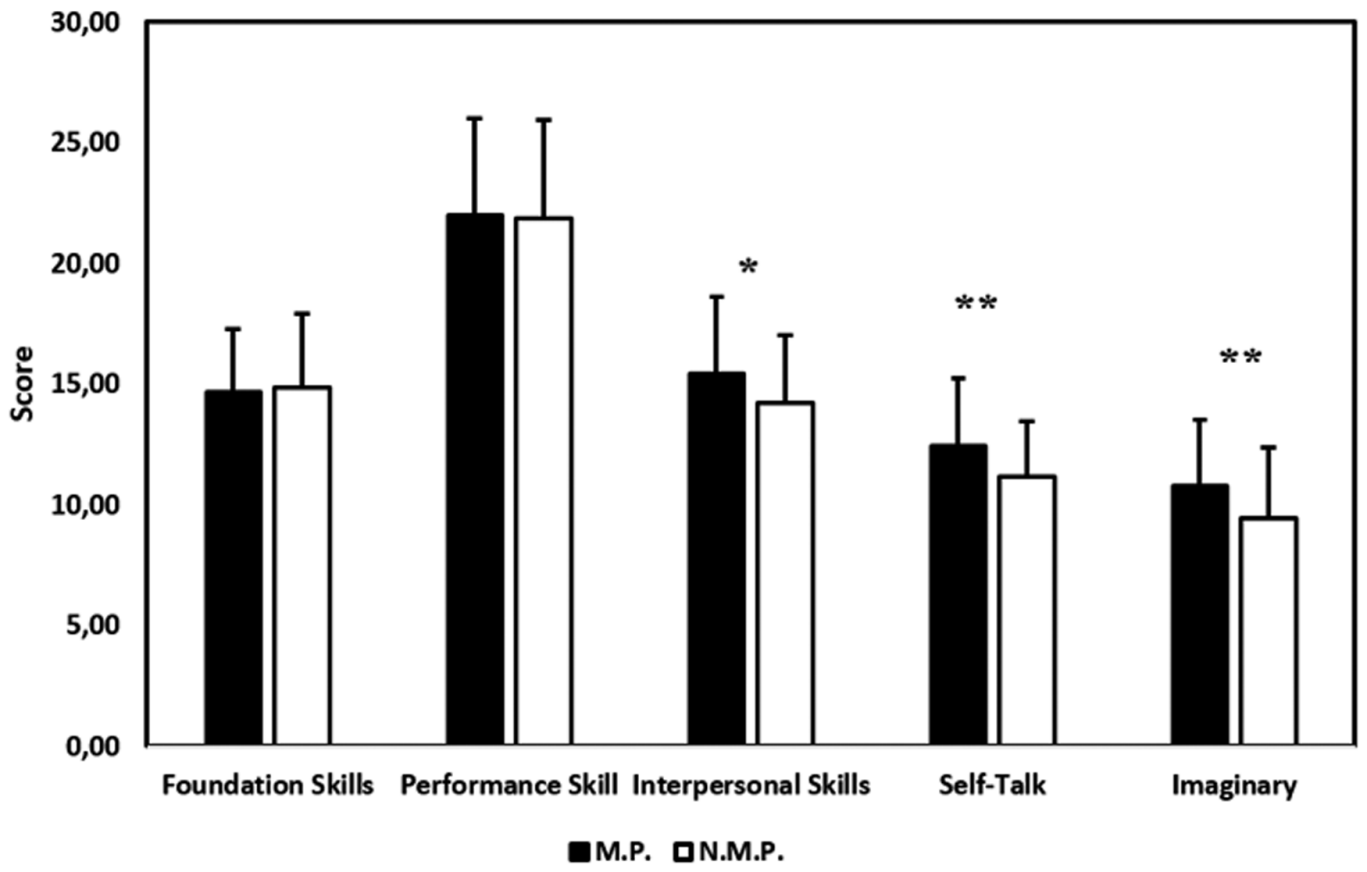
Comparison of mental preparation (M.P.) and No Mental Preparation (N.P.) groups. * indicates *p* < 0.05; ** indicates *p* < 0.01.

The effect sizes (Cohen's *d*) were calculated for the significant results. The Interpersonal Skills effect size was *d* = 0.39, indicating a medium effect. For Self-Talk, the effect size was *d* = 0.49, and for Imaginary Skills, *d* = 0.47, both reflecting medium effects. Finally, for the Total Score, the effect size was *d* = 0.37, also suggesting a medium effect size. The differences between athletes who engage in mental preparation and those who do not are illustrated in Figure 2.

**Figure 3:**
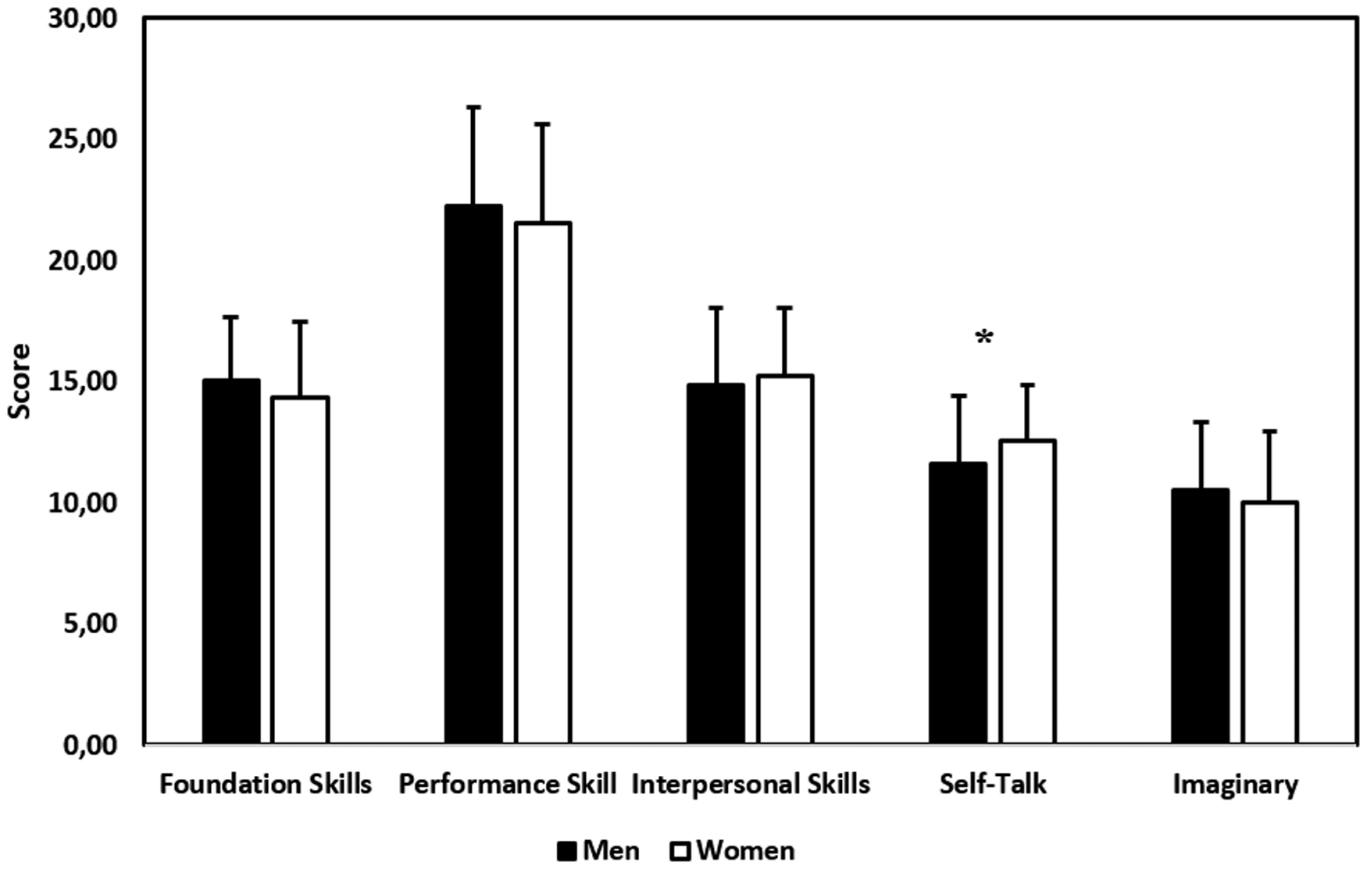
Comparison of gender differences. *indicates *p* < 0.05; ** indicates *p* < 0.01.

Gender differences were tested using an independent samples *t*-test. The results indicated significant differences in the Self-Talk category, revealing a notable gender effect [*t*(199) = −2.61, *p* = 0.011], where men (*M* = 11.62, *SD* = 2.77) scored lower than women (*M* = 12.55, *SD* = 2.28), resulting in a medium effect size (Cohen's *d* = −0.36). In contrast, no significant differences were found in the Foundation Skills [*t*(199) = 1.696, *p* = 0.091], Performance Skills [*t*(199) = 1.244, *p* = 0.215], Interpersonal Skills [*t*(199) = −1.018, *p* = 0.310], Imaginary Skills [*t*(199) = 1.311, *p* = 0.191], or Total Score [*t*(199) = 0.365, *p* = 0.715] categories. These findings suggest that the Self-Talk intervention had a notable impact, particularly regarding gender differences, while the other skills did not exhibit statistically significant differences (see Figure 3).

### Characteristics of mental and sports tactical preparation

To investigate the techniques employed during mental training, athletes were prompted with the question, “What mental preparation techniques do you use, if any?” Respondents were allowed to select multiple techniques. The most frequently reported method was Mental Training, utilized by 73 participants (36%). This category encompassed Inner Mental Training, Psychological Skills Training, goal-setting, and imagination exercises. The second most common approach was Relaxation Techniques, indicated by 56 participants (27%), which included Autogenic Training, Jacobson's Progressive Relaxation Technique, breathwork techniques, and biofeedback. Mindfulness-based techniques were identified by 47 respondents (23%). Less commonly employed techniques included Attention and Concentration Techniques (e.g., Thought-Stopping technique, Flashlight technique, and keyword utilization), reported by 39 respondents (19%), Cognitive Techniques, utilized by 16 respondents (7%), and Psychotherapeutic Techniques (e.g., Systematic Desensitization, Neuro-linguistic Programming), used by 5 respondents (2%). Additionally, general psychological and life coaching emerged as a prominent method, reported by 65 participants (32%).

Regarding the mental training approaches employed, 65 athletes indicated that they did not engage in any form of structured mental training (Group N.M.P.). Seventy-nine athletes reported self-directed preparation without professional oversight. Of the remaining participants, 37 trained independently with the support of their running coach, 7 worked under the guidance of a mental skills trainer, and 13 sought the expertise of a sports psychologist. Notably, no participants reported engaging in mental training within a group setting.

Regarding tactical preparation for races, athletes were asked, “How do you prepare for the tactical specifics? These can include pace changes and the tactics of potential opponents”. The most frequent responses indicated that 104 participants (51%) practiced different tempo changes during training, while 51 participants (25%) engaged in various tactical behaviors during preparatory races, such as leading or running in a pack. Furthermore, video analysis and the examination of race split times and positional data were conducted by 74 participants (36%). Additionally, 57 respondents (27%) reported preparing for tactical challenges during mental preparation. Participants could provide multiple responses to this question.

## Discussion

A key finding of this study is the validation of the Sport Mental Training Questionnaire, which demonstrated good internal consistency (69). This establishes it as a reliable tool for assessing mental preparedness in distance runners and facilitates better monitoring of changes in training effects. The focus on a shorter questionnaire that includes universal components applicable across multiple sports may be particularly useful in early testing phases, group assessments, or situations with limited trainer time resources (70).

The Sports Mental Training Questionnaire (SMTQ) serves as a valuable complement to existing assessment tools by providing a focused measure of mental skills—key psychological attributes that significantly influence athletic performance. While Mahoney's Psychological Skills Inventory for Sport (PSIS) evaluates broader constructs such as motivation, self-confidence, concentration, mental preparation, and anxiety control (71), it may not thoroughly explore the specific mental skills that can be developed through targeted training. Similarly, Orlick's work emphasizes commitment, belief, and mental readiness, primarily concentrating on distraction control and constructive evaluation (72), yet it may not offer a systematic approach to assessing the nuanced aspects of mental skills. Bull and colleagues’ Mental Skills Questionnaire expands on this by evaluating imagery, mental preparation, self-confidence, relaxation, and anxiety management (73), but it may not fully address the importance of pinpointing specific mental attributes that can be directly influenced through training. The Test of Performance Strategies (TOPS) provides insights into goal-setting, emotional control, and attentional strategies (68, 74), while the Ottawa Mental Skills Assessment Tool (OMSAT-3) integrates foundational, psychosomatic, and cognitive skills, including imagery and competition planning (75). Additionally, the Psychological Performance Inventory – A (PPI-A) by Golby emphasizes determination and positive cognition (76), but it may not specifically isolate mental skills for targeted development. In this context, the SMTQ fills a vital niche by providing a comprehensive measure of mental preparedness in distance runners, facilitating a nuanced understanding of how specific mental skills influence performance outcomes. Its brevity and reliability enhance its practicality for both sports psychologists and coaches, allowing for efficient assessment and the development of targeted interventions that effectively address athletes’ unique psychological needs, ultimately optimizing performance in competitive sports.

This study also highlights critical aspects of mental preparation among distance runners, particularly focusing on gender differences. The results indicate that female participants reported significantly higher levels of Self-Talk compared to their male counterparts (77, 78). This finding may align with previous research suggesting that women tend to use different strategies, such as relying more on internal dialogue to overcome obstacles, which may enhance their mental resilience during competitions (45, 52, 79). Furthermore, athletes participating in mental training scored significantly higher in both mental techniques (Self-Talk and Imagery), Interpersonal Skills and Total Scores, supporting the hypothesis that mental preparation can be critical to success (71, 80). In terms of competition level, the study did not find significant differences based on event types or competition levels. This finding contrasts with existing literature, which suggests that elite athletes engage in more advanced mental preparation strategies (67, 81). This discrepancy may be attributed to non-directly guided mental learning during preparation (82–84).

Adequate sport psychology training and monitoring of current preparedness are particularly important in high-stress situations, such as middle-distance running races characterized by uncertain outcomes and frequent tactical competitions with multiple pace shifts. In these contexts, high concentration, along with emotional and decision-making response readiness, significantly influences success (24, 85–87). Furthermore, marathons and longer ultra-distance races present challenges associated with extreme durations. In these longer races, the literature indicates that selecting the appropriate pace can prevent the phenomenon of “hitting the wall”, which is associated with a sudden onset of very high fatigue occurring in the last third of the distance (27, 88–90). In these situations, mental techniques like positive self-talk, mental reframing, and focusing on external or internal objects can help overcome challenges (42, 91). Additionally, various other sport psychology techniques can help manage pre-competition anxiety and cope with post-competition results, thereby enabling athletes to maintain their mental well-being and remain engaged in the sport (92).

Group sport psychology sessions in which runners learn to apply performance enhancement skills could reach more people, thus improving athletes’ sports performance and enjoyment of sports and mental well-being (93). Examples of such closed-ended group intervention techniques include REBT or Mindfulness-based interventions (e.g., MSPE) (94, 95). The latter emphasizes a heightened sense of “meta-awareness” or impartial contemplation of negative thoughts, which are often effectively utilized today, as it facilitates not only emotion and attention regulation but also the flow state (96–98). In this context, assessing and monitoring athletes’ mental abilities can be highly rewarding. Shorter questionnaires that focus on critical components facilitate easier repeated measurements and enhance monitoring of training effects. The Sport Mental Training Questionnaire is a concise, valid instrument suitable for measuring the mental preparation of distance runners. The findings indicate good internal consistency across all subscales, making it effective for quick and repeatable assessments of mental preparedness.

## Strengths of the research

One of the primary strengths of this study lies in its large sample size, which includes a substantial number of elite athletes competing at both national and international levels. This is particularly noteworthy, as similar research typically focuses solely on amateur athletes, making this study a rare and valuable contribution. The diversity of the participants enhances the external validity of the findings, allowing for broader generalizations regarding mental and tactical preparation among long-distance runners. To the best of our knowledge, this is the first study to validate a tool specifically designed to assess both the tactical and mental skills of runners, offering critical insights into the dynamics of mental readiness across various competitive contexts.

## Limitations

This study has several limitations that may affect the interpretation of the findings. The reliance on self-reported data introduces the potential for bias, as participants might overestimate their use of mental preparation techniques; however, anonymity was ensured to encourage honest responses. Additionally, the cross-sectional design restricts the ability to establish causal relationships between mental preparation and performance outcomes. Future research could benefit from longitudinal studies to address this issue.

## Further research directions

Future research opportunities should focus on testing tools to measure tactical preparation and awareness, as well as assessing the effectiveness of various training methods, including individual, mentored, and sports psychologist-led approaches. Furthermore, exploring the impact of cognitive and mindfulness-based group interventions presents a promising area for further study, as these group interventions can reach a broader audience. Ultimately, this research contributes to the growing body of literature that emphasizes the critical role of mental preparation in athletic performance, encouraging coaches and sports psychologists to integrate psychological training into their athletes’ preparation regimens.

## Conclusion

The investigation into the mental preparation of distance runners and the associated assessment tools remains underexplored. Our research demonstrated that the 20-item Sports Mental Training Questionnaire serves as a reliable and efficient measure of distance runners’ mental capabilities and their influence on performance. A significant finding was that mental preparedness did not differ across gender, competition level, or event; however, sport psychological training had a notable positive impact. Women were more inclined to use internal dialogue techniques. Limitations of this questionnaire-based study include potential self-report biases, as participants might exaggerate their use of mental preparation techniques or their perceived readiness. Furthermore, the cross-sectional design limits the ability to establish causal relationships between mental preparation and performance outcomes. The Sports Mental Training Questionnaire is a concise and validated instrument for assessing the mental skills essential for success, as well as for evaluating the effectiveness of interventions aimed at developing these skills during the training process.

## Data Availability

The raw data supporting the conclusions of this article will be made available by the authors, without undue reservation.
